# Impact of the antimicrobial stewardship program on hospital-acquired candidemia

**DOI:** 10.1038/s41598-022-19374-3

**Published:** 2022-09-07

**Authors:** Yoshiro Hadano, Asuka Suyama, Ayako Miura, Shigeo Fujii, Yoshiko Suzuki, Yoshitaka Tomoda, Yukikazu Awaya

**Affiliations:** 1 Itabashi Chuo Medical Center, Itabashi-ku, Tokyo Japan; 2 Division of Infection Control and Prevention, Shimane University Hospital, Izumo, Shimane Japan; 3 Department of Pharmacy, Itabashi Chuo Medical Center, Itabashi-ku, Tokyo Japan; 4 Department of Clinical Laboratory, Itabashi Chuo Medical Center, Itabashi-ku, Tokyo Japan; 5 Department of Nursing, Itabashi Chuo Medical Center, Itabashi-ku, Tokyo Japan; 6 Department of Medicine, Division of General Medicine, Itabashi Chuo Medical Center, Itabashi-ku, Tokyo Japan; 7 Division of Pulmonary Medicine, Department of Medicine, Itabashi Chuo Medical Center, Itabashi-ku, Tokyo Japan

**Keywords:** Medical research, Diseases, Infectious diseases

## Abstract

Antibiotic stewardship programs reduce antibiotic use without negative clinical outcomes. However, epidemiological data describing the relationship between implementing antimicrobial stewardship and candidemia incidence are scarce. This study aimed to evaluate the effect of antibiotic stewardship on the incidence of hospital acquired candidemia. We conducted a retrospective study from April 2017 to September 2020. We reviewed patients that were treated with three broad-spectrum antipseudomonal agents: carbapenem, tazobactam/piperacillin, and cefepime. Monthly aggregated hospital antimicrobial consumption was measured as days of therapy (DOTs) per 1000 patient-days, and the monthly incidence of hospital acquired candidemia was recorded. The median monthly carbapenem-DOTs during pre-intervention and intervention were 28.4 and 10.0, respectively. Time-series analysis showed significant level changes after intervention: − 10.0 DOTs (*p* = 0.02). There was a downward trend in the monthly carbapenem-DOTs after intervention. The median hospital-acquired candidemia incidence was 0.17 and 0.08 per 1000 patient-days during pre-intervention and intervention periods, respectively. Time-series analysis showed a significant level change after intervention (− 0.16 per 1000 patient-days; *p* = 0.048). The trend in the incidence of hospital-acquired candidemia did not significantly change between pre-intervention and intervention. Decreased broad-spectrum antibiotic use (particularly carbapenem) by our antimicrobial stewardship term may reduce hospital-acquired candidemia incidences.

## Introduction

Antimicrobial resistance (AMR) has become a global concern, and promoting appropriate use of antimicrobial agents is an urgent issue. Implementation of AMR-prevention measures is an urgent requirement for medical institutions^1^.

Infections caused by resistant microorganisms, such as fungal infections caused by *Candida* spp., are associated with increased mortality^2^. Inappropriate use of antimicrobial agents is thought to be an important factor in increasing the incidence of these infections^3^. Antimicrobial stewardship program (ASP) intervention in a tertiary care facility reduced the use of broad-spectrum antimicrobials without negative outcomes^4,5^. However, the specific indicators of clinical outcomes, including the incidence of bacteremia, remains unknown. Although antibiotic pressure is one of the risk factors for *Candida* spp.^6,7^, its relationship to candidemia incidence is not well established. Activities implemented by the Antimicrobial Susceptibility Team (AST) at Itabashi Chuo Medical Center (ICMC) in April 2018 resulted in a downward trend of broad-spectrum antimicrobial use. This study evaluated whether the reduction of broad-spectrum antimicrobial use through the implementation of antimicrobial stewardship led to a deceased incidence of hospital-acquired blood stream infections (BSIs) due to *Candida* spp. in an acute tertiary care hospital in Japan.

## Methods

This single-center retrospective study was conducted at ICMC, which is a 569-bed tertiary community-based acute care teaching hospital and teaching center with 34 subspecialties and a 12-bed intensive care unit, in Tokyo, Japan. The number of ambulance transports per year is approximately 10,000. This hospital has an active Department of General Internal Medicine, and its average number of inpatients is approximately 80 per day. This department manages cases with a variety of medical problems, including infectious diseases. The Department of Transplant Surgery is also active, and performs about 40 solid organ transplants per year, mainly kidney transplants. The study was approved by the Institutional Review Board of Itabashi Chuo Medical Center (No. 220125B). The requirement to obtain written consent from all participants was waived by the Institutional Review Board because of the study’s observational nature without any deviation from the current medical practice.

The impact of our ASP on antimicrobial use and the incidence of hospital-acquired candidemia, in the inpatient setting, was evaluated between two periods (pre-intervention: April 2017 to March 2018 and intervention: April 2018 to September 2020). This data were retrospectively collected from electronic charts.

A multidisciplinary antimicrobial stewardship program was implemented in April 2018 (Table 1). It was staffed by three physicians (respiratory medicine, pediatrics, and nephrology; 0.1 full-time equivalent), a clinical pharmacist (1.0 full-time equivalent), a microbiology laboratory technician (0.1 full-time equivalent), an infection control nurse (0.1 full-time equivalent), and a part-time infectious diseases physician (0.1 full-time equivalent). AST members held a 1.5 h case conference once a week. Inclusion criteria were defined as: patients treated with three broad-spectrum antipseudomonal agents (carbapenems, tazobactam/piperacillin, and cefepime) and intravenous quinolones for more than 7 days, having positive blood cultures, and being unresponsive to treatment. The monthly aggregated hospital antimicrobial consumption was measured as days of therapy (DOTs) per 1000 patient-days for the broad-spectrum agents. The monthly incidence of hospital acquired candidemia was measured between April 2017 and September 2020. The data used in this study were obtained from medical charts. All episodes of hospital-acquired BSIs due to *Candida* spp. were retrospectively reviewed by AST members.

**Table 1:** Preintervention and intervention activities in Itabashi Chuo Medical Center.

Pre-intervention period (From April 2017 to March 2018)	Intervention period (From April 2018 to September 2020)
1. Monitoring antimicrobial use density (carbapenems, tazobactam/piperacillin, cefepime, intravenous quinolones, vancomycin, daptomycin, and linezolid) 2. Therapeutic drug monitoring (vancomycin)	1. Monitoring antimicrobial use density (carbapenems, tazobactam/piperacillin, cefepime, intravenous quinolones, vancomycin, daptomycin and linezolid) 2. Therapeutic drug monitoring (vancomycin) 3. Weekly 1.5 h case conference by antimicrobial stewardship team members indication criteria - Patients treated with broad-spectrum antipseudomonal agents for more than 7 days (carbapenems, tazobactam/piperacillin, cefepime, and intravenous quinolones) - Positive blood culture - Unresponsive to antibiotic treatment 4. Infectious diseases consultations by part-time infectious diseases specialist (once a week)

### Definitions

The total DOTs per month per 1000 patient-days was calculated for carbapenem (CAR), and 3-antipseudomonal agents: carbapenem (meropenem), piperacillin–tazobactam, and cefepime. Meropenem was evaluated because its consumption was more than 98% of all carbapenems during this period. Hospital-acquired BSIs due to *Candida* spp. were diagnosed based on blood culture specimens obtained after 48 h since admission. Only the first episode of BSIs during the study period was included, but a separate episode could be recognized if there was another episode of hospital-acquired BSI that occurred 30 days after completing a course of antifungal therapy. The monthly incidence of candidemia was calculated per 1000 patient-days.

### Statistical analysis

Interrupted time series (ITS) regression analysis was used to evaluate trends in monthly antimicrobial consumption and hospital-acquired candidemia before and after intervention. DOTs per 1,000 patient-days and trends in the monthly CAR DOT, 3-antipseudomonal DOT, and incidence of detected hospital-acquired candidemia were used^8^. Changes were evaluated with an ordinary least-squares regression. Harmonic terms were added to the models to account for seasonality. Categorical data were analyzed using either the chi-squared test or Fisher’s exact test and non-categorical data using the Student’s t-test or Wilcoxon rank sum test, as appropriate. A two-tailed *p*-value of < 0.05 was regarded as statistically significant, and the 95% confidence interval (CI) was used. All statistical analyses were performed using the R software version 4.0.2. (The R Foundation for Statistical Computing, Vienna, Austria) and SAS software version 9.4 (SAS Institute, Cary, NC, USA).

## Results

There was a total of 502,132 hospital admissions at ICMC, including 146,287 hospitalizations in the pre-intervention period (12,191 average hospitalization per month) and 355,845 hospitalizations (11,862 average hospitalizations per month) in the intervention period. The number of blood cultures per 1000 patients was 68.2 and 66.3 in the baseline period and intervention period, respectively. A total of 50 cases of hospital-acquired candidemia were analyzed, and 26 and 24 cases were included in the pre-intervention and intervention periods, respectively. During the study period, *Candida albicans* was observed in 11 patients (42.3%) in the pre-intervention period and in 11 patients (45.8%) in the intervention period (Table 2). There were no significant differences between the two groups (*p* = 0.72). The number of hospital-acquired candidemia cases in the intensive care unit (ICU) were five cases (19.2%) and three cases (12.5%), respectively (*p* = 0.51).

**Table 2:** Baseline characteristics of patients with hospital–acquired candidemia between the pre-intervention and intervention periods.

Variables	Pre-intervention period		Intervention period		*P* value
	n = 26	Percent (%)	n = 24	Percent (%)	
**Setting**					
Intensive care unit at the time of blood culture collection	5	19.2	3	12.5	0.51
**Microbiology**					0.72
*C. albicans*	11	42.3	11	45.8	
*C. glabrata*	11	42.3	7	29.2	
*C. tropicalis*	2	7.7	2	8.3	
*C. parapsilosis*	2	7.7	2	8.3	
*C. lusitaniae*	0	0	2	8.3	

### Trends in carbapenem antibiotic use

**Figure 1:**
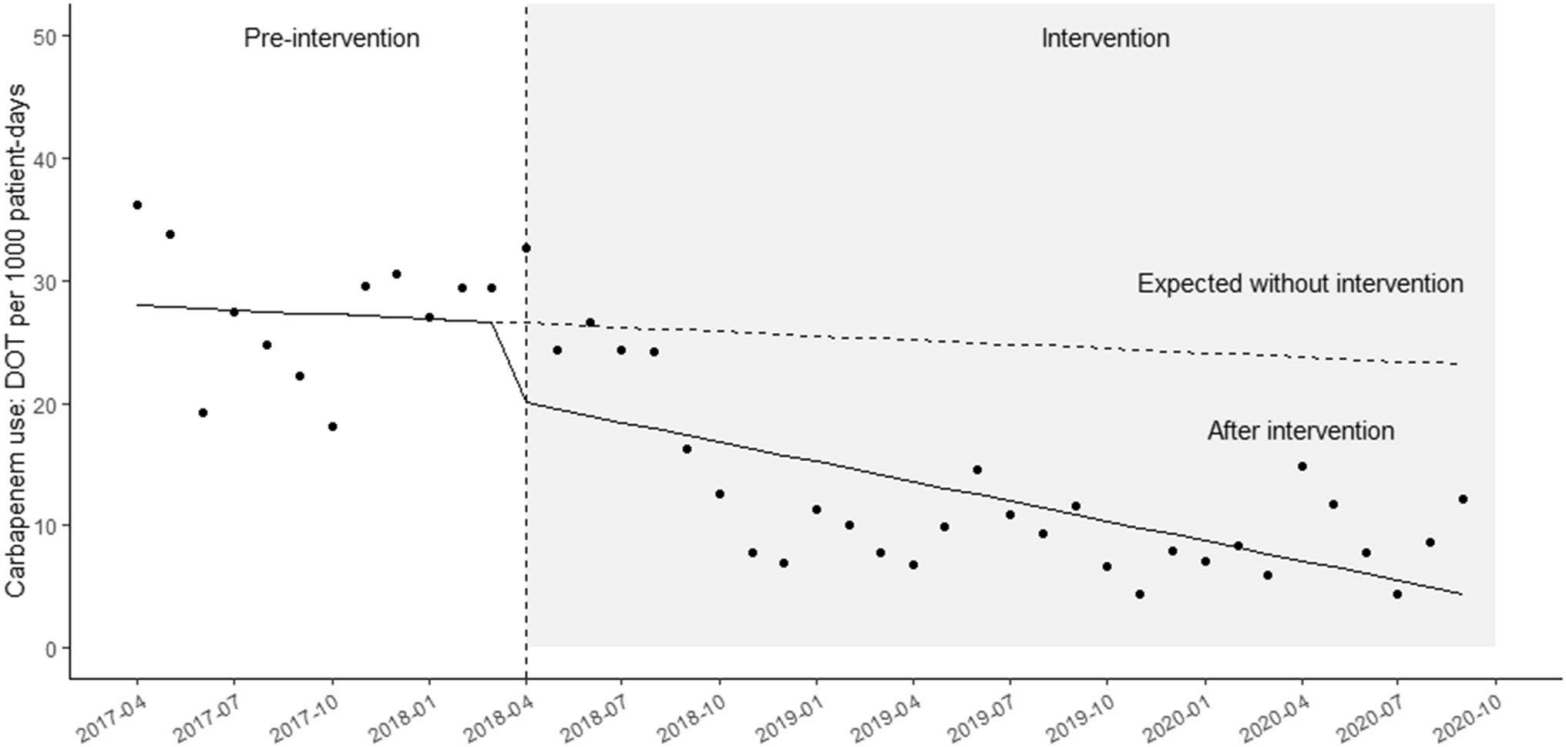
Time series analysis of monthly antibiotic use. Time series plot showing the monthly average duration of carbapenem use (DOTs/1000 patient-days).

The median monthly CAR-DOT was 28.4 (interquartile range [IQR], 18.1 to 30.0) and 10.0 (IQR, 7.8 to 14.6) per 1000 patient-days per during the pre-intervention period and intervention period, respectively (*p* < 0.01). The time-series analysis showed a significant change in levels after the intervention: − 10.0 DOTs per 1000 patient-days (95% confidence interval [CI], − 19.0 to − 2.0; *p* = 0.02; Fig. 1). A downward trend was observed in the monthly CAR-DOT during the intervention period (coefficient: − 0.54; 95% CI: − 0.77 to − 0.30, *p* < 0.01; Fig. 1).

### Trends using 3-antipseudomonal antibiotics

**Figure 2:**
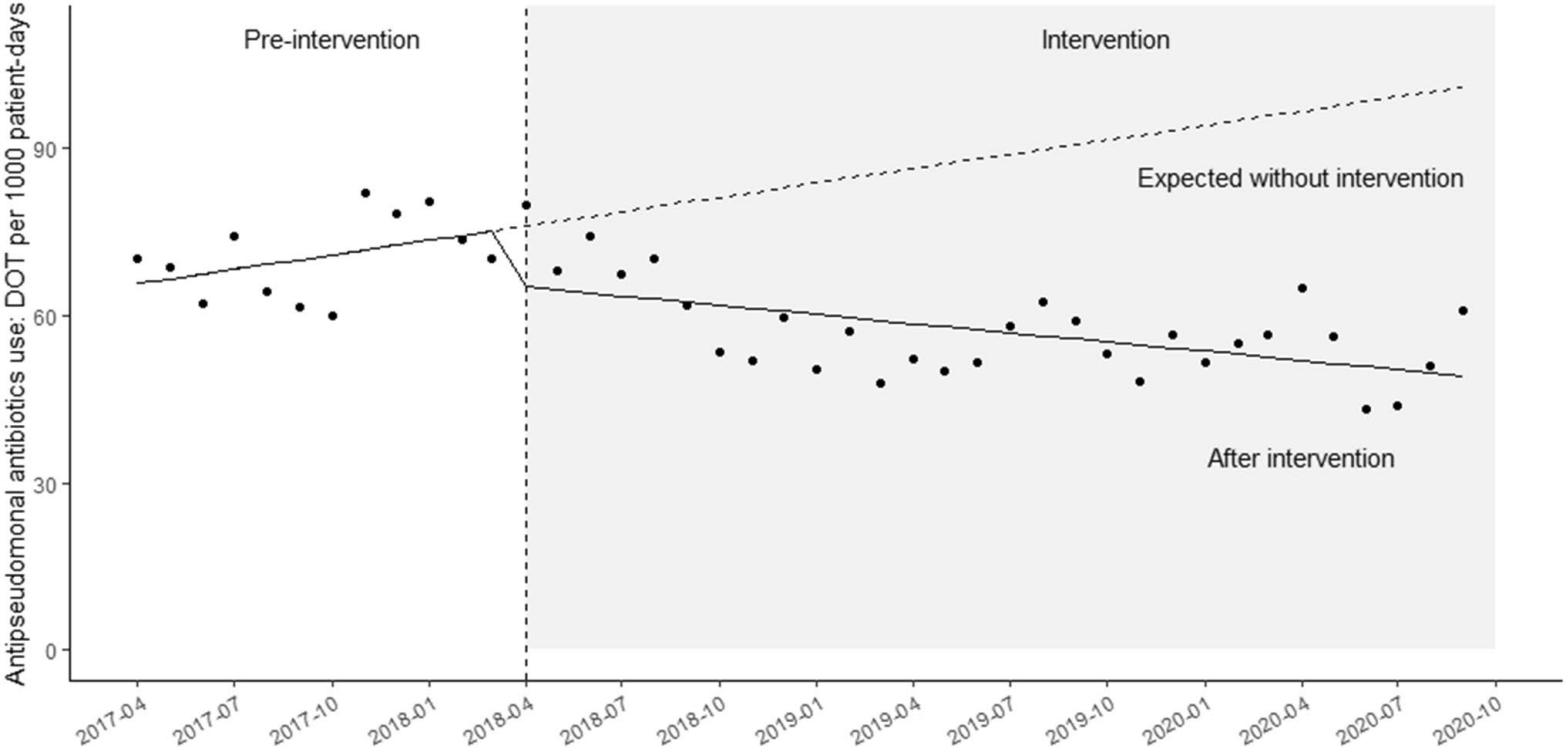
Time series analysis of monthly antibiotic use. Time series plot showing the monthly average duration (DOTs/1000 patient-days) of use for three antipseudomonal antibiotics: carbapenem, tazobactam/piperacillin, and cefepime.

The median monthly DOT for the 3-antipseudomonal antibiotics was 69.4 (IQR, 61.9 to 69.7) and 56.3 (IQR, 51.6 to 62.4) per 1000 patient-days during the pre-intervention and intervention periods, respectively (*p* < 0.01). The time-series analysis showed that there was a significant level change after the intervention: − 14.0 DOTs per 1000 patient-days (95% CI, − 25.0 to − 2.5; *p* = 0.02; Fig. 2). There was an upward trend in the monthly DOTs of 3-antipseudomonal agents during the pre-intervention period (coefficient: 1.6; 95% CI: 0.29 to 3.0, *p* = 0.02), while a downward trend was observed in monthly DOTs for antipseudomonal agents (coefficient: − 0.66; 95% CI: − 1.0. to − 0.36, *p* < 0.01) after the intervention (Fig. 2).

### Incidence of hospital-acquired candidemia

**Figure 3:**
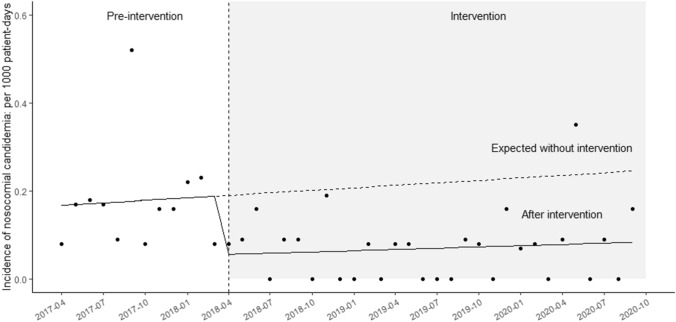
Time series analysis of candidemia incidence. Time series plot showing the impact of antimicrobial use on the incidence density (per 1000 patient-days) of hospital-acquired candidemia.

The median incidence of hospital-acquired candidemia was 0.17 (IQR, 0.09 to 0.18) and 0.08 (IQR, 0.0 to 0.09) per 1000 patient-days during the pre-intervention and intervention periods, respectively (*p* < 0.01). Time-series analysis showed that there was a significant level change after intervention: − 0.16 per 1,000 patient-days (95% CI, − 0.31 to − 0.00; *p* = 0.048; Fig. 3). The trend in the incidence of hospital-acquired candidemia did not significantly change between the pre-intervention period (coefficient: 0.00; 95% CI: − 0.01 to 0.02, *p* = 0.60) and the intervention period (coefficient: 0.00; 95% CI: − 0.00 to 0.00, *p* = 0.70).

In the sub-analysis in the intensive care unit (ICU) setting, the median incidence of hospital-acquired candidemia in the ICU at the time of blood culture collection was 0.00 (IQR, 0.00 to 0.09) and 0.00 (IQR, 0.0 to 0.00) per 1000 patient-days during the pre-intervention and intervention periods, respectively (*p* = 0.04).

## Discussion

The decrease in broad-spectrum antibiotic use (especially carbapenems) achieved a sustained clinical impact by reducing the incidence of hospital-acquired candidemia. This was attributed to the education-based antimicrobial stewardship program which was effective in decreasing the incidence and mortality rate of hospital-acquired candidemia and multidrug-resistant infections^9^. However, several ASP studies did not show a statistically significant reduction in the incidence of hospital-acquired candidemia with a reduction in broad-spectrum antimicrobial use^10–12^. In this study, the relatively long study period and the decrease in the use of antimicrobials (especially CAR-DOT) may have contributed to the decrease in the incidence of hospital-acquired *Candidemia*.

The mortality of candidemia with treatment is approximately 25% to 40%^13,14^, while treatment delay increases its occurrence^15^. Preventing the progression of candidemia is important for reducing mortality and for economic reasons associated with prolonged hospitalization. The mean total cost per patient with candidemia and IC ranges from $48 487 to $157 574, whereas the mean cost of hospitalization per patient with candidemia and IC is between $10 216 and $37 715^16^. This study showed that candidemia development can be decreased by reducing the use of broad-spectrum antimicrobials through the ASP.

This study has several limitations related to its retrospective and uncontrolled nature. First, it was conducted at a single center; thus, the study findings may not be generalizable to other settings. Second, this study did not measure the 30-day mortality of hospital-acquired candidemia and other hospital-acquired multidrug-resistant BSIs, such as extended spectrum β-lactamase- (ESBL) producing or carbapenem resistant organisms. The impact of other interventions, including improved hand hygiene, infection control, catheter related blood stream infection (CRBSI), a more detailed analysis on *Candida* spp., such as antifungal consumption, and resistance ratio between the periods was not considered in this study. However, there were no outbreaks of CRBSI in the study period, and specific infection control interventions to prevent the incidence of CRBSI were not performed. A future multicenter study with a large number of clinical cases is needed to elucidate the relationship between the administration of broad-spectrum antibiotics and the incidence of hospital-acquired candidemia and multidrug resistant bacteremia.

In summary, a reduction in broad-spectrum antibiotic administration (especially carbapenem) by our AST reduced the incidence rate of hospital-acquired candidemia in an acute tertiary care hospital.

## Data Availability

The datasets used and/or analyzed in the current study are available from the corresponding author upon request.
